# Epithelioid glioblastoma presenting as aphasia in a young adult with ovarian cancer: A case report

**DOI:** 10.1002/ccr3.2088

**Published:** 2019-03-18

**Authors:** Megan M. Finneran, Joseph Georges, Michael Kakareka, Ryan Moncman, Miriam Enriquez, Todd Siegal, Gregory Kubicek, Alan Turtz, Steven Yocom, H. Warren Goldman, James Barrese

**Affiliations:** ^1^ Department of Clinical Medicine Chicago College of Osteopathic Medicine Chicago Illinois; ^2^ Department of Neurosurgery Philadelphia College of Osteopathic Medicine Philadelphia Pennsylvania; ^3^ Department of Neurosurgery Cooper University Hospital Camden New Jersey; ^4^ Department of Pathology Cooper University Hospital Camden New Jersey; ^5^ Department of Radiology Cooper University Hospital Camden New Jersey; ^6^ Department of Radiation Oncology Cooper University Hospital Camden New Jersey

**Keywords:** epithelioid, glioblastoma, IDH wild type, multiforme

## Abstract

Our patient's clinical history and preoperative radiographic evaluation suggested central nervous system (CNS) metastatic disease. Ultimately, final pathology revealed epithelioid glioblastoma (eGBM), a newly classified CNS primary tumor. This reinforces the importance of direct tissue sampling and including eGBM on the differential for young patients with histories of systemic cancer presenting with new CNS lesions.

## 1 INTRODUCTION

Epithelioid glioblastoma (eGBM) is a rare, recent addition to the World Health Organization's (WHO) classification of central nervous system (CNS) tumors. We describe a unique case of a young woman with a history of ovarian cancer presenting with expressive aphasia and a new left temporal brain lesion. Open biopsy provided a diagnosis of eGBM. Here, we discuss this unique case and a relevant literature review of eGBM.

## 2 CASE REPORT

### 2.1 History

A 29‐year‐old woman with past medical history of breast fibroadenoma and ovarian juvenile‐type granulosa cell tumor at age 14 presented to the emergency room following two episodes of disorientation, global aphasia, and left‐sided facial and lower extremity numbness with paresthesias. The patient did not lose consciousness, was somnolent after both episodes but recovered, and was able to continue with her work that day. Further history revealed the patient had experienced intermittent headaches during the prior 2 weeks. She denied trauma, seizure history, vision changes, gait instability, or recent illness.

Patient had a left salpingo‐oophorectomy at age 14. Routine follow‐up scans for 5 years after her cancer diagnosis suggested remission. Her last menstrual period was 2 days prior to presentation. Social history revealed the patient drank socially, never smoked, and she denied drug use. Patient's mother had a cerebral vascular abnormality and her maternal aunt died of a brain aneurysm. The patient's father was diagnosed with rectal cancer at age 37 and died of T‐cell lymphoma at age 47. Her paternal grandfather had colon and lung cancer at age 47 and died at age 50. Her maternal grandmother was diagnosed with multiple myeloma at age 78.

### 2.2 Examination

Vital signs: Temp 99.0ºF, BP 125/77 mm Hg, Pulse 98/min, RR 18/min, SpO2 99%, BMI 31.76 kg/m^2^. She was in no acute distress and cooperative throughout the examination. The patient was alert and oriented to self, time, and place with no focal neurological deficits.

### 2.3 Diagnostic imaging, testing, and laboratory results

**Figure 1:**
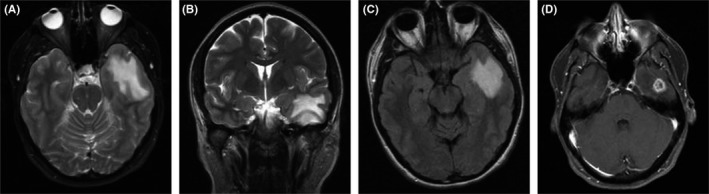
Preoperative MRI. A, Axial and B, coronal T2 MRI demonstrated a hyperintense left temporal lobe lesion. C, Perilesional hyperintensity on FLAIR sequence suggestive of vasogenic edema. D, Postcontrast T1 MRI revealed a solitary enhancing left temporal lobe lesion

MRI brain with and without contrast demonstrated an intra‐axial anterior left temporal lobe enhancing mass measuring 1.5 cm in diameter. The lesion was isointense on T1‐weighted images, hyperintense on T2‐weighted images, showed ring enhancement on T1 with contrast and had perilesional hyperintensity on FLAIR sequence suggestive of vasogenic edema (Figure 1). A 21 channel continuous digital EEG performed at bedside yielded no epileptiform activity.

Given the patient's history and imaging, a metastatic workup was conducted. Relevant laboratory results are highlighted in Table 1. CT chest, abdomen, and pelvis were remarkable for a 1.4 cm × 2.2 cm soft tissue density within the left breast consistent with a benign fibroadenoma. A transvaginal ultrasound was performed which demonstrated a left salpingo‐oophorectomy and a right ovary that measured 4 cm × 2.8 cm × 3.7 cm with slightly complex cysts that measured 2.1 cm × 1.5 cm × 2.2 cm, consistent with physiologic corpus luteal cysts.

**Table 1:** Relevant laboratory results

Tumor marker	Patient value	Normal range
CA‐125	17 U/mL	<35 U/mL
CEA	1.4 ng/mL	0‐5.0 ng/mL
Inhibin A	4 pg/mL	<98.0 pg/mL
Inhibin B	32 pg/mL	<153 pg/mL

### 2.4 Hospital course and operation

The patient was admitted to the oncology service with frequent neurological evaluations and started on dexamethasone 4 mg every 6 hours and levetiracetam 500 mg twice daily. Five days after admission, the patient underwent an image‐guided left frontal‐temporal craniotomy. The patient's head was secured in a radiolucent skull clamp and an intra‐operative CT scan obtained in the surgical position was fused to a preoperative thin slice postcontrast MRI, and a surgical navigation system was registered to the patient with good accuracy. She underwent a left temporal craniotomy where the subcortical tumor was localized with image guidance and was found to be firm and well encapsulated. The lesion was removed *en bloc* and sent for frozen and permanent pathology. Frozen section suggested a poorly differentiated malignant metastatic lesion of unknown primary origin.

A postoperative MRI scan within 24 hours of surgery demonstrated gross total resection (Figure 3A). She remained neurologically intact and was discharged to home on post‐op day number 2 on a steroid taper.

### 2.5 Pathologic findings

**Figure 2:**
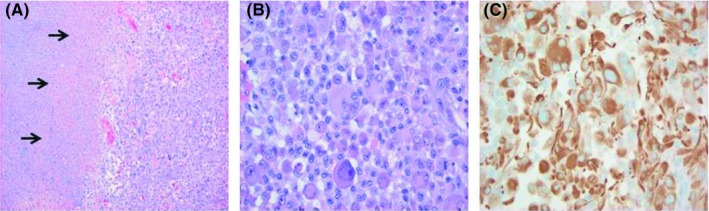
Histology. A, Areas of necrosis are appreciated under low magnification H&E staining (arrows; 40×). B, Epithelioid tumor cells showing plump eosinophilic cytoplasm and sharp cell borders, mimicking metastatic melanoma or carcinoma (400×). C, Tumor cells expressed GFAP by immunohistochemistry, supporting glial origin (400×)

Histologic sections revealed tumor with large areas of necrosis and numerous mitotic figures (Figure 2A). The section showed predominantly pleomorphic epithelioid cells showing plump eosinophilic cytoplasm and sharp cell borders mimicking a metastatic melanoma or carcinoma (Figure 2B). The morphologic features were inconsistent with an ovarian juvenile‐type granulosa cell tumor. Immunohistochemistry showed that the tumor was positive for GFAP, supporting glial differentiation (Figure 2C). Immunostains for melanoma including SOX10, HMB45, and S100 were negative. An immunostain for p53 also showed diffuse positivity and INI‐1 showed no loss of nuclear expression. A reticulin stain revealed absence of an intercellular reticulin meshwork. Molecular analyses were also obtained and revealed no mutations in the IDH1 and IDH2 genes (wild‐type) and in exon V600 of the BRAF gene. Mutations for EGFR variant III expression and MGMT promoter methylation were also negative. The overall findings were consistent with glioblastoma in which the epithelioid type was supported by the cytologic features—namely the sharp cell borders and rounded nuclei—that imparted a more epithelioid, rather than glial, appearance.

### 2.6 Outcome

**Figure 3:**
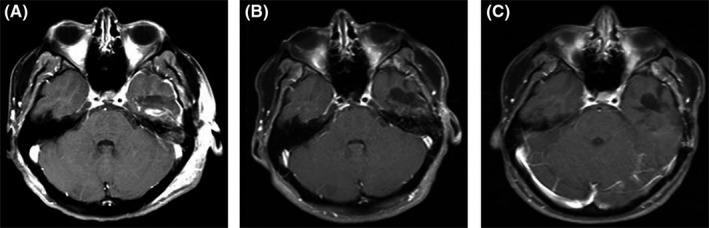
Postoperative T1 postcontrast MRI. A, 24‐hour postoperative MRI reveals gross total resection with a signal in the posterior resection bed consistent with blood products and proteinaceous fluid. B, 3‐mo and C, 6‐mo postoperative MRIs show T1 hypointensity in left temporal lobe without areas of residual or new enhancement

Four weeks after surgery, the patient underwent fractionated partial brain irradiation consisting of 60 Gy over 6 weeks. She completed an MRI brain with and without contrast at 3 and 6 months postoperatively which showed no evidence of tumor recurrence (Figure 3). Patient has since refused adjuvant temozolomide therapy and is currently seeking nutritional therapies. She has been seizure‐free on levetiracetam throughout her clinical course and remains neurologically intact.

## 3 DISCUSSION

Glioblastoma epithelioid type is a rare tumor. It was added to the 4th edition of the World Health Organization Classification of Tumors of the Central Nervous System in 2016.^1^ According to this classification, glioblastomas are largely divided based on isocitrate dehydrogenase‐1 (IDH‐1) mutations.^2^ IDH wild type, or primary glioblastoma, develops de novo without evidence of a less malignant precursor. These tumors compose about 90% of cases and are often seen in elderly patients.^2^ IDH mutant, or secondary glioblastoma, progresses from low‐grade astrocytomas and accounts for about 10% of cases. Secondary gliomas are more often seen in younger patients, frequently in the frontal lobe and carry a better prognosis.^2^ Histologically, the two subgroups are mostly indistinguishable with standard stains and the diagnosis relies on genetic identification of the IDH‐1 mutation.

The epithelioid type has been added to the classification of IDH wild‐type GBM, but it is unique in its predilection for younger patients and poorer prognosis.^1^ The poor prognosis may be in part due to an increased propensity for leptomeningeal dissemination.^3^ Diagnosis of eGBM relies on a combination of radiologic, histologic, and genetic analyses. Radiologic features of eGBM include areas of cystic necrosis with nodular enhancement, often accompanied by vasogenic edema and associated mass effect.^4^ Tumor location and morphology can assist in differentiating primary brain tumors from solitary intracerebral metastases with contrast‐enhanced MRI. Metastases are typically located at gray‐white matter junctions and are well‐circumscribed compared to primary tumors. The lesion in this case resided within temporal lobe parenchyma and was relatively well‐circumscribed, providing a differential which included both primary brain tumor and metastasis.^5^ Though MR spectroscopy and MR perfusion may assist differentiating primary brain tumors from solitary brain metastases, these MR sequences were not obtained for this case.^6^, ^7^

Genetically, eGBM differs from traditional IDH wild‐type glioblastomas in that it often lacks identifiers such as EGFR amplification.^1^ Histologically, the differential diagnosis includes metastatic carcinoma, metastatic melanoma, and pleomorphic xanthoastrocytoma (PXA).^4^, ^8^ Epithelioid GBM features large epithelioid cells with abundant eosinophilic cytoplasm, vesicular chromatin, multiple mitotic figures, fairly extensive necrosis, and prominent nucleoli. The appearance of the nucleoli particularly may cause eGBM to be mistaken for metastatic melanoma during frozen section analysis. Its histology may also closely resemble PXA, a WHO grade II lesion, and its anaplastic variant (PXA‐A) as both often exhibit pleomorphic and epithelioid cell morphology.^8^ Distinguishing features that are expected in PXA, which were notably absent in the presented case, include presence of eosinophilic globules (eosinophilic granular bodies) and a rich reticulin meshwork. Immunohistochemical stains assist in narrowing the differential further, as GBM is typically GFAP‐positive, S‐100 protein‐positive, negative for HBM‐45 and Melan‐A. Metastatic melanoma is also S‐100 protein‐, HMB‐45‐, and Melan‐A‐positive, but metastatic melanomas and carcinomas are GFAP‐negative.^4^ Our case was immunostain‐negative for S‐100 and SOX10. This is in agreement with reports showing 29% of eGBMs are negative for S‐100 and reports showing SOX10 is not expressed in all GBM samples.^9^, ^10^ Molecularly, an increasing number of eGBM cases are showing a BRAF V600E mutation.^11^ The BRAF mutation could provide clinical relevance, as BRAF inhibitor therapy may become a promising option.^12^

## 4 SUMMARY

Identification and diagnosis of glioblastoma are often straightforward; however, eGBM can mimic a metastatic tumor radiographically and on frozen section. Therefore, establishing a definitive histologic diagnosis may be important in selected patients suspected of having metastatic disease. Given the unique classifications of GBM, it is important to evaluate brain tumor specimens using histologic stains and genetic analysis for detection of eGBM. Due to its poor prognosis and predilection for leptomeningeal dissemination, early identification and treatment of this lesion may improve overall patient outcomes. Here, we discuss a 29‐year‐old woman with past medical history of ovarian cancer presenting with transient receptive aphasia and radiographic evidence of a temporal lesion resembling metastatic disease. Although the initial impressions on frozen section appeared consistent with a metastasis, final histologic and genetic analysis of this lesion revealed eGBM, a rare and newly classified subtype of GBM by the WHO classification.

## CONFLICT OF INTEREST

None declared.

## AUTHOR CONTRIBUTION

MMF: conceived idea; acquired data; prepared manuscript. JG: conceived idea; acquired data; prepared manuscript; supervised project. MK and RM: acquired data and prepared the manuscript. ME: analyzed and interpreted data; edited manuscript; analyzed and interpreted histopathologic data. TS and GK: analyzed and interpreted data; edited manuscript; analyzed and interpreted radiographic data. AT, SY, and HWG: analyzed and interpreted data and edited the manuscript. JB: conceived idea; analyzed and interpreted data; prepared manuscript; edited manuscript; and supervised project.

## References

[1] Louis DN , Perry A , Reifenberger G , et al. The 2016 World Health Organization classification of tumors of the central nervous system: a summary. Acta Neuropathol. 2016;131(6):803‐820. 27157931 10.1007/s00401-016-1545-1

[2] Ohgaki H , Kleihues P . The definition of primary and secondary glioblastoma. Clin Cancer Res. 2013;19(4):764‐772. 23209033 10.1158/1078-0432.CCR-12-3002

[3] Sugimoto K , Ideguchi M , Kimura T , et al. Epithelioid/rhabdoid glioblastoma: a highly aggressive subtype of glioblastoma. Brain Tumor Pathol. 2016;33(2):137‐146. 26667174 10.1007/s10014-015-0243-3

[4] Liebelt BD , Boghani Z , Takei H , et al. Epithelioid glioblastoma presenting as massive intracerebral hemorrhage: case report and review of the literature. Surg Neurol Int. 2015;6(Suppl 2):S97‐S100. 25883856 10.4103/2152-7806.153643 PMC4392545

[5] Blanchet L , Krooshof PW , Postma GJ , et al. Discrimination between metastasis and glioblastoma multiforme based on morphometric analysis of MR images. AJNR Am J Neuroradiol. 2011;32(1):67‐73. 21051512 10.3174/ajnr.A2269 PMC7964969

[6] Eguia Del Valle A , Lopez‐Vicente J , Martinez‐Conde R , et al. Current understanding of genetic polymorphisms as biomarkers for risk of biological complications in implantology. J Clin Exp Dent. 2018;10(10):e1029‐e1039. 30386510 10.4317/jced.55141 PMC6203903

[7] Tsougos I , Svolos P , Kousi E , et al. Differentiation of glioblastoma multiforme from metastatic brain tumor using proton magnetic resonance spectroscopy, diffusion and perfusion metrics at 3 T. Cancer Imaging. 2012;12:423‐436. 23108208 10.1102/1470-7330.2012.0038 PMC3494384

[8] Alexandrescu S , Korshunov A , Lai SH , et al. Epithelioid glioblastomas and anaplastic epithelioid pleomorphic xanthoastrocytomas‐same entity or first cousins? Brain Pathol. 2016;26(2):215‐223. 26238627 10.1111/bpa.12295 PMC8029361

[9] Ferletta M , Uhrbom L , Olofsson T , et al. Sox10 has a broad expression pattern in gliomas and enhances platelet‐derived growth factor‐B–induced gliomagenesis. Mol Cancer Res. 2007;5(9):891‐897. 17855658 10.1158/1541-7786.MCR-07-0113

[10] Khanna G , Pathak P , Suri V , et al. Immunohistochemical and molecular genetic study on epithelioid glioblastoma: series of seven cases with review of literature. Pathol Res Pract. 2018;214(5):679‐685. 29615337 10.1016/j.prp.2018.03.019

[11] Kleinschmidt‐DeMasters BK , Aisner DL , Birks DK , et al. Epithelioid GBMs show a high percentage of BRAF V600E mutation. Am J Surg Pathol. 2013;37(5):685‐698. 23552385 10.1097/PAS.0b013e31827f9c5e PMC4610349

[12] Chapman PB , Hauschild A , Robert C , et al. Improved survival with vemurafenib in melanoma with BRAF V600E mutation. N Engl J Med. 2011;364(26):2507‐2516. 21639808 10.1056/NEJMoa1103782 PMC3549296

